# Cascading phase transformations in high carbon steel resulting in the formation of inverse bainite: An atomic scale investigation

**DOI:** 10.1038/s41598-019-42037-9

**Published:** 2019-04-03

**Authors:** Rangasayee Kannan, Yiyu Wang, Jonathan Poplawsky, Sudarsanam Suresh Babu, Leijun Li

**Affiliations:** 1 Department of Chemical and Materials Engineering, University of Alberta, Edmonton, Alberta T6G 2V4 Canada; 2 Materials Science and Technology Division, Oak Ridge National Laboratory, Oak Ridge, TN 37831-6064 USA; 3 Center for Nanophase Materials Sciences, Oak Ridge National Laboratory, PO Box 2008 MS 6064, Oak Ridge, TN 37831-6064 USA; 4 Department of Mechanical, Aerospace and Biomedical Engineering, University of Tennessee, Knoxville, TN 37996 USA

## Abstract

Atom probe tomography (APT) has been used to understand the redistribution of solutes during the isothermal cascading phase transformations from supersaturated austenite resulting in the formation of inverse bainite. Different cascading reactions resulting in the formation of inverse bainite, namely the cementite midrib formation, ferrite formation, secondary cementite formation, and the degenerated microstructure of inverse bainite have been studied in detail. Solute profiles across the different transformation interfaces indicate Negligible Partitioning Local Equilibrium (NPLE) type growth kinetics for cementite midrib, whereas a transition in growth kinetics from Para Equilibrium (PE) to Negligible Partitioning Local Equilibrium (NPLE) is observed for secondary cementite and ferrite transformation. The results provide a strong indication that the inverse bainitic transformation occurs as a consequence of individual cascading phase transformations starting from parent austenite, and the transformation of inverse bainite occurs in a similar manner to Widmanstatten ferrite/bainitic ferrite with carbon diffusion-controlled growth, and without any reconstructive or long-range diffusion of substitutional solutes.

## Introduction

The transformation of bainite is the most studied phase transformation in steels. Since bainite forms in an intermediate temperature between the reconstructive pearlite/ferrite transformation and the displacive martensitic transformation, two opposing theories have been associated with the bainitic transformation^1–7^. The first theory, also called the ledgewise theory of bainite, states that bainitic transformation is essentially a reconstructive transformation progressing by the thermally assisted movement of atoms^2,6,7^. The second theory is that bainitic transformation is entirely displacive in nature, similar to martensitic transformation, with atomic movement less than the inter-atomic spacing^1,3–5^. Extensive evidences for supporting both the diffusional nature of bainitic transformation and the displacive nature of bainitic transformation can be found in the literature. Since bainitic transformation occurs at an intermediate temperature range between the reconstructive pearlite/ferrite and dislpacive martensite, the morphology and the carbide precipitation sequence in bainite varies with the transformation temperature of bainite. Upper and lower bainite are the two established terms explaining this variation in the morphology and the carbide precipitation sequence in bainite, with upper bainite forming at a higher temperature close to the pearlite transformation, and lower bainite forming at a lower temperature close to the transformation temperature of martensite. There are, however, a number of other morphologies of bainite, including granular bainite, columnar bainite, alloy pearlite, coalesced bainite, spiky pearlite, and inverse bainite^8,9^. Bhadeshia has reviewed the different morphologies of bainite and the formation mechanism in ref.^8^. The formation mechanism of these different morphologies of bainite can be explained using either the ledgewise theory of bainite transformation or the displacive theory of bainite transformation. Summarizing extensive research spanning over the period of decades and studying different transformation characteristics, a systematic set of transformation characteristics exhibited by upper bainite, lower bainite, and Widmanstätten ferrite are summarized in ref.^8^.

A unique morphology of bainite occurring in hypereutectoid steels is called the inverse bainite. The term was coined by Hillert in 1957^10^, where he observed a symmetry in the iron-carbon system with respect to the carbon content. Hillert hypothesized that much like how bainite forms by the nucleation of ferrite being the first nucleation event from austenite at lower carbon content, and pearlite forming by a cooperative growth between ferrite and cementite near the eutectoid carbon concentration, there must be a third transformation product with cementite nucleation being the first nucleation event from parent austenite at higher carbon concentrations. Hillert named this microstructure with cementite being the lead nucleating phase from parent austenite as inverse bainite. Since Hillert’s proposal, the term inverse bainite has been used sparsely in the literature, with the main focus on proving the existence of inverse bainite through micrographs^11–14^. Much recent studies of inverse bainite include those by Kolmskog and Borgenstam^15–17^, where they studied the eutectoid transformations in a 4 wt.% Cr hypereutectoid steel. Inverse bainite was used as an evidence for the bainitic transformation being a diffusional-displacive controlled transformation. Goulas *et al*.^18^ reported the formation of inverse bainite in a martensitic matrix in chromium enriched regions of a hypoeutectoid steel. We have carried out studies on the microstructural evolution resulting in the formation of inverse bainite^19–21^ during isothermal holding in a hypereutectoid steel and found that the transformation of inverse bainite proceeds by the formation of cementite as the lead nucleating phase from parent austenite acting as the midrib for the microstructure. This is followed by ferrite formation from carbon depleted regions of the cementite midrib/austenite interface (the second stage). The nucleation of cementite in ferrite on either side of the cementite midrib (named secondary cementite) is the third stage. Lastly, the conversion of the entire inverse bainitic unit to upper bainite, named the degeneration of inverse bainite is the final stage of the transformation. Since the majority of the transformation characteristics of bainite and Widmanstätten ferrite listed in ref.^8^ require a sub-nm scale analysis of the transformation interfaces, we have used Atom Probe Tomography (APT) to understand the redistribution of solutes during the progress of inverse bainitic transformation during isothermal holding in a hypereutectoid steel. Considering the multi-stage progress of the transformation, different transformation interfaces, namely the cementite midrib formation, ferrite formation, secondary cementite formation, and the formation of the degenerated microstructure of inverse bainite have been studied in detail at an atomic scale for the first time.

## Results

### Microstructure of inverse bainite

**Figure 1:**
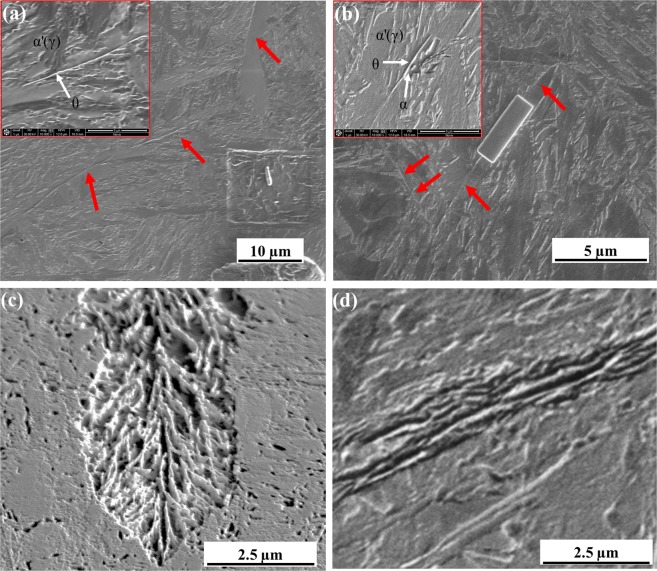
Microstructural evolution during isothermal bainite transformation. (**a**) Secondary electron micrograph of the 1 minute isothermal hold sample. (**b**) Secondary electron micrograph of the 3 minutes isothermal hold sample. (**c**) Secondary electron micrograph of the 5 minutes isothermal hold sample, and (**d**) Secondary electron micrograph of the 10 minutes isothermal hold sample. Prior austenite grain boundary are indicated by red arrows in (**a**,**b**).

Representative microstructural evolution during the isothermal bainitic transformation are shown in Fig. 1. Figure 1a shows the microstructure of the 1 minute (cementite midrib formation) isothermal hold sample indicating the formation of carbide along prior austenite grain boundary. Figure 1b shows the microstructure of the 3 minutes isothermal hold sample representing the formation of ferrite surrounding the cementite. When the transformation time is increased to 5 minutes, carbides within ferrite nucleate as shown in Fig. 1c. Figure 1c represents inverse bainite microstructure with carbide midrib (formed during 1 minute into the transformation), ferrite surrounding the carbide midrib (formed during 3 minutes into the transformation), and secondary carbides within ferrite surrounding the carbide midrib (formed during 5 minutes into the transformation). For the 10 minutes isothermal hold sample in Fig. 1d, carbide midrib can no longer be identified and the microstructure represents an upper-bainitic type microstructure. Atom probe needle were site specifically lifted-out from these locations, and the atom probe reconstructions are discussed next.

### Solute redistribution during inverse bainitic cementite midrib transformation

**Figure 2:**
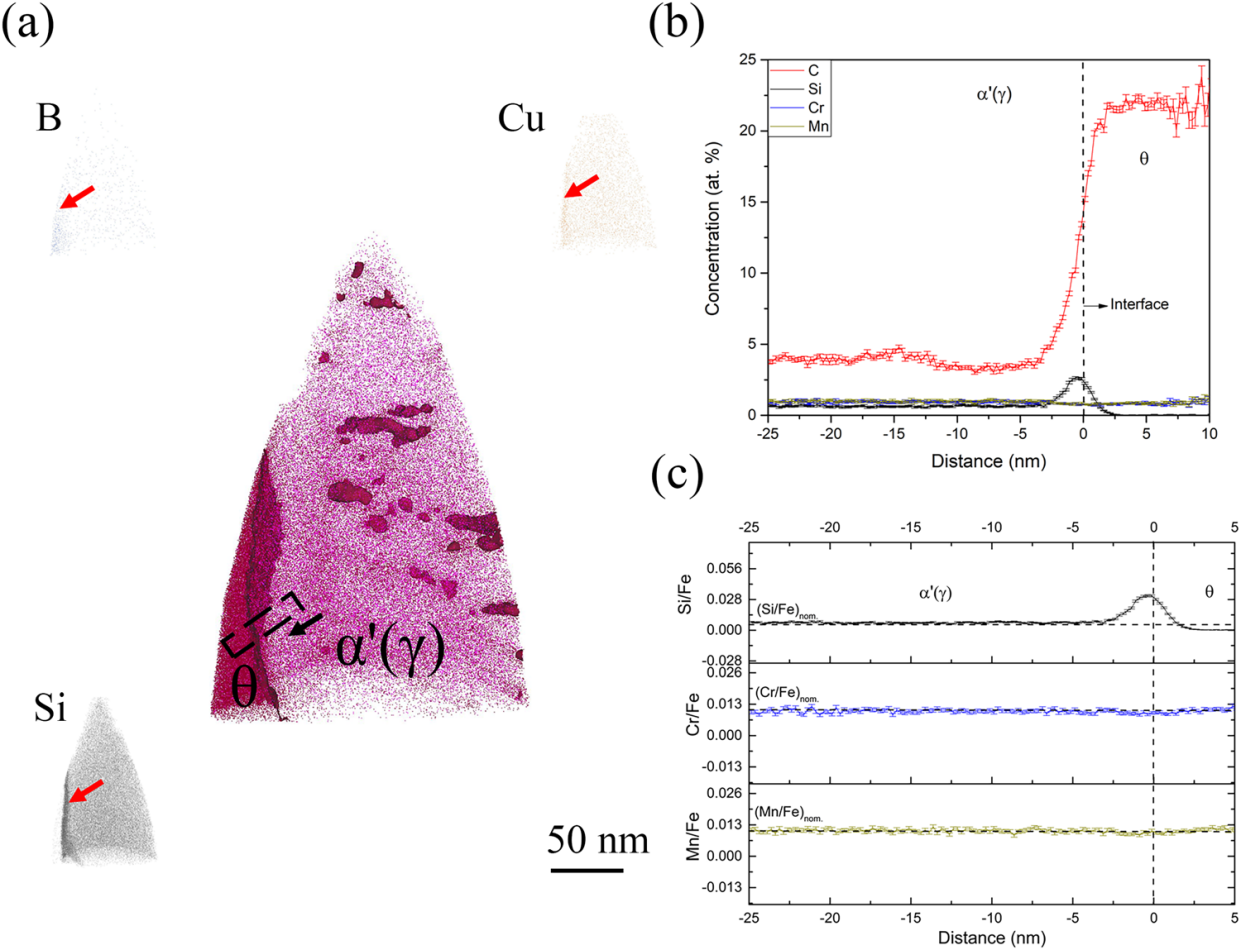
APT reconstructions across the cementite/martensite interface in the 1 minute isothermal hold sample. (**a**) Atom maps of C, B, Cu, and Si. (**b**) Proximity histogram across the cementite/martensite interface showing the solute redistribution, (**c**) Ratio of substitutional solute concentration to iron concentration (Cr/Fe and Mn/Fe) across the cementite/martensite interface.

**Figure 3:**
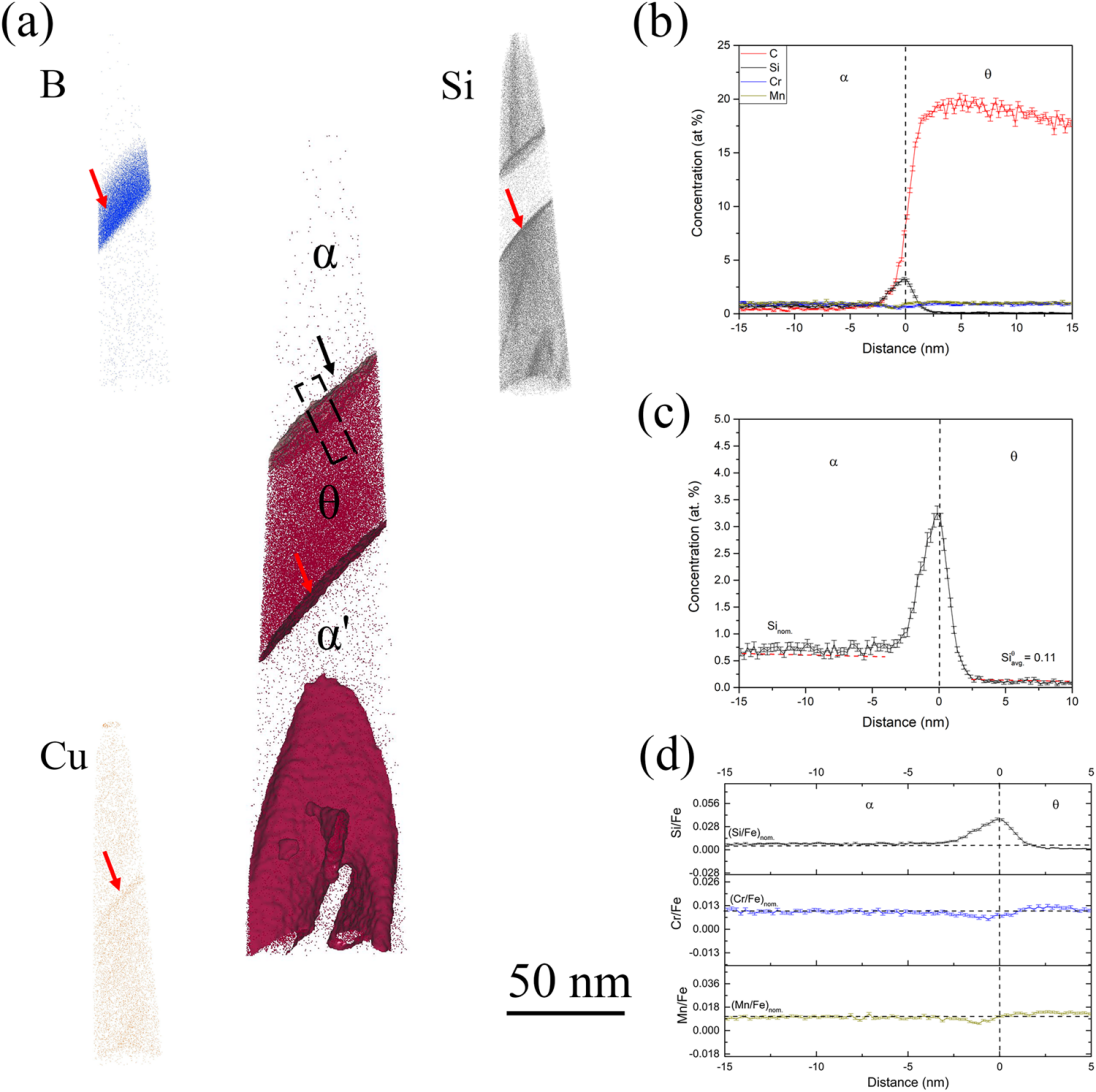
APT reconstructions across cementite/ferrite interface in the 3 minute isothermal hold sample. (**a**) Atom maps of C, B, Cu, and Si. (**b**) Proximity histogram across the cementite/ferrite interface showing the solute redistribution, (**c**) Proximity histogram across the cementite/ferrite interface showing the Si redistribution across the interface (**d**) Ratio of substitutional solute concentration to iron concentration (Cr/Fe and Mn/Fe) across the cementite/ferrite interface.

Figure 2 shows the APT results of the 1 minute isothermal hold sample, representing the formation of inverse bainitic cementite in a martensitic matrix. From the carbon atom map, a carbon enriched carbide region is observed. Away from the carbide, clusters with approx. 15 at.% C are observed in the martensite. The carbon concentration of cluster is similar to the ones reported by Peet^22^ and Caballero^23,24^. The high sensitivity of the LEAP allows for identification and visualization of the trace elements like B, Cu, and Si at the carbide/martensite interface. Proximity histograms across the carbide/martensite interface, and the ratio of substitutional solutes to iron across the interface are shown in Fig. 2b,c respectively. It can be seen that the carbide has a carbon concentration of around 25 at.%, martensite has a carbon concentration of around 4 at.%, which is the nominal carbon concentration of the steel. The carbon concentration of the carbide suggests that the carbide is cementite. Si concentration in cementite is almost 0 at.%, whereas martensite has a nominal Si concentration of 0.4 at.%. A Si concentration spike is observed at the cementite/martensite interface. The concentration of Cr and Mn are 1 at.% in both martensite and cementite, and no partitioning of Cr/Mn is observed at the interface. It can also be seen that the ratio of Cr/Fe as well as Mn/Fe is maintained across the cementite/martensite interface, confirming that no long range diffusion of substitutional solutes takes place. To further understand the redistribution of substitutional solutes during cementite midrib formation, the cementite midrib/inverse bainitic ferrite was imaged in the atom probe using the 3 minute isothermal hold sample. The results of the cementite midrib/ferrite interface are shown in Fig. 3. Figure 3a shows the C, B, Si, and Cu atom maps. From the carbon atom map, a carbon enriched carbide region is observed. Below the carbide, martensitic matrix with carbon clusters (cementite in martensite) is observed, and above the carbide, inverse bainitic ferrite (low carbon region) is observed. At the cementite/martensite interface, segregation of Si, Cu, and B is observed. In order to characterize the cementite/ferrite interface, proximity histograms were reconstructed. Figure 3b shows the redistribution of solutes across the cementite midrib/inverse bainitic ferrite. It can be seen that the carbide has a carbon concentration of around 25 at.% indicating that the carbide is cementite, and ferrite has a much lower carbon concentration of 0.5 at.%. No change in the Cr/Mn concentration is observed across the cementite/ferrite interface, and both cementite and ferrite have nominal concentration of Cr and Mn (1 at.% each). Figure 3c displays the proximity histogram for Si across the cementite/ferrite interface. It can be seen that cementite has some Si retained in it (around 0.1 at.%, which is much greater than the equilibrium solubility of Si in cementite) whereas the concentration of Si in ferrite is the nominal Si content (around 0.5 at.%). The ratio of Cr/Mn to iron shown in Fig. 3d confirms that no long range diffusion of Cr/Mn takes place across the transformation interface.

### Solute redistribution during inverse bainitic ferrite transformation

**Figure 4:**
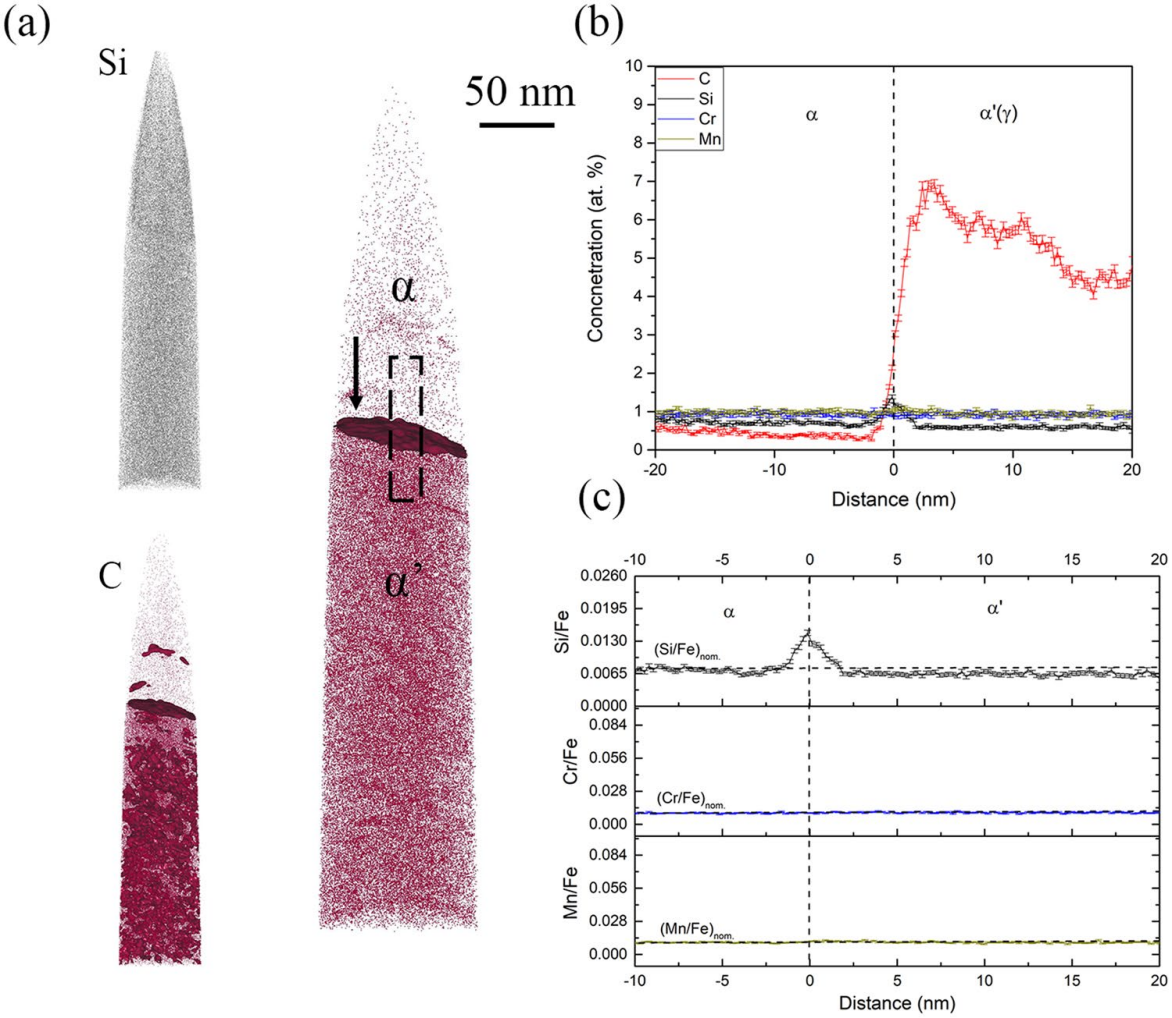
APT reconstructions across the ferrite/martensite interface in the 5 minute isothermal hold sample. (**a**) Atom maps of C, Si, 3 at.% iso-concentration surface showing the low/high carbon interfaces in the APT datasets. (**b**) Proximity histogram across the ferrite/martensite interface showing the solute redistribution, (**c**) Ratio of substitutional solute concentration to iron concentration across the ferrite/martensite interface.

**Figure 5:**
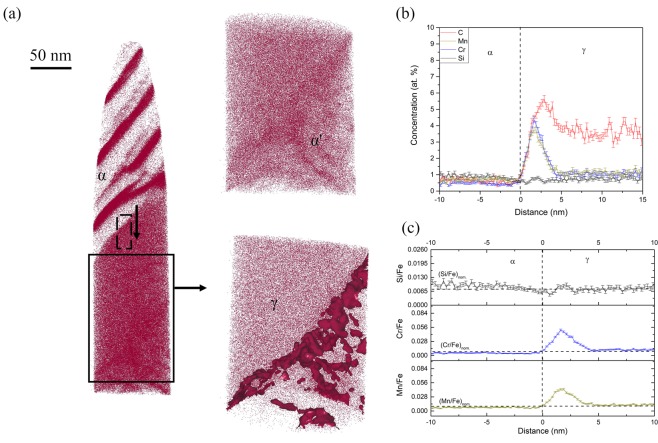
APT reconstructions across the ferrite/martensite(retained austenite) interface in the 10 minute isothermal hold sample. (**a**) C atom map for the complete data set of the APT experiment and a clipped region containing the austenite/martensite interface (8 at.% iso-concentration surface). (**b**) Proximity histogram across the ferrite/austenite interface showing the solute redistribution, (**c**) Ratio of substitutional solute concentration to iron concentration across the ferrite/austenite interface.

The 5 minutes and 10 minutes isothermal hold samples were used for the analysis of growth kinetics of inverse bainitic ferrite. APT results across the ferrite/martensite interface of the 5 minutes and 10 minutes isothermal hold samples are shown in Figs 4 and 5 respectively. Figure 4a displays the carbon atom map, Si atom map, and the 3 at.% iso-concentration surface showing all the interfaces for the 5 minutes isothermal hold sample. Figure 4b displays the proximity histogram showing the solute redistribution across the ferrite/martensite interface. C redistribution can be seen across the martensite/ferrite interface. As for the substitutional solutes are concerned, no partitioning of Cr/Mn is observed. The Cr/Mn concentration is the nominal concentration (1 at.% each) in both ferrite and martensite. Si redistribution with concentration spike is observed at the interface. Figure 4c displays the ratio of substitutional solute to iron atoms across the ferrite martensite interface. The ratio of substitutional solute to iron is preserved, indicating that no long range diffusion of substitutional solutes takes place across the interface. Figure 5a displays C atom map for the 10 minutes isothermal hold sample. Alternating layers of secondary carbides (carbon rich regions) distributed in inverse bainitic ferrite (carbon depleted regions) can be seen. Below the ferrite, high carbon martensite/retained austenite region is observed. A 8 at.% iso-concentration surface of the martensite/retained austenite region close to the inverse bainitic ferrite delineates austenite (free from carbon clusters) from martensite (with carbon clusters with carbon concentration of approx. 15 at.% similar to the ones reported by Peet^22^ and Caballero^23,24^). Figure 5b displays the proximity histogram across the ferrite/retained austenite interface. The carbon concentration in ferrite is low, whereas the carbon concentration in austenite close to the interface is around 6 at.%. Away from the ferrite/austenite interface, the carbon concentration in austenite reaches the nominal carbon concentration of 4 at.%. It can also be seen from Fig. 5b that a Cr/Mn concentration spike with a width of around 4 nm is observed at the interface, with the concentration reaching the nominal concentration of 1 at.% each away from the concentration spike. Unlike the 5 minute isothermal hold sample in Fig. 4, Si does not partition across the interface. The ratio of substitutional solutes to iron shown in Fig. 5c shows that the Si/Fe ratio is preserved across the interface, indicating no long range diffusion of Si. Cr, Mn concentration spike is evident, but the Cr/Fe and Mn/Fe ratio is preserved away from the interface, indicating no long range diffusion of Cr/Mn across the transformation interface.

### Solute redistribution during the formation of secondary carbides in inverse bainite

**Figure 6:**
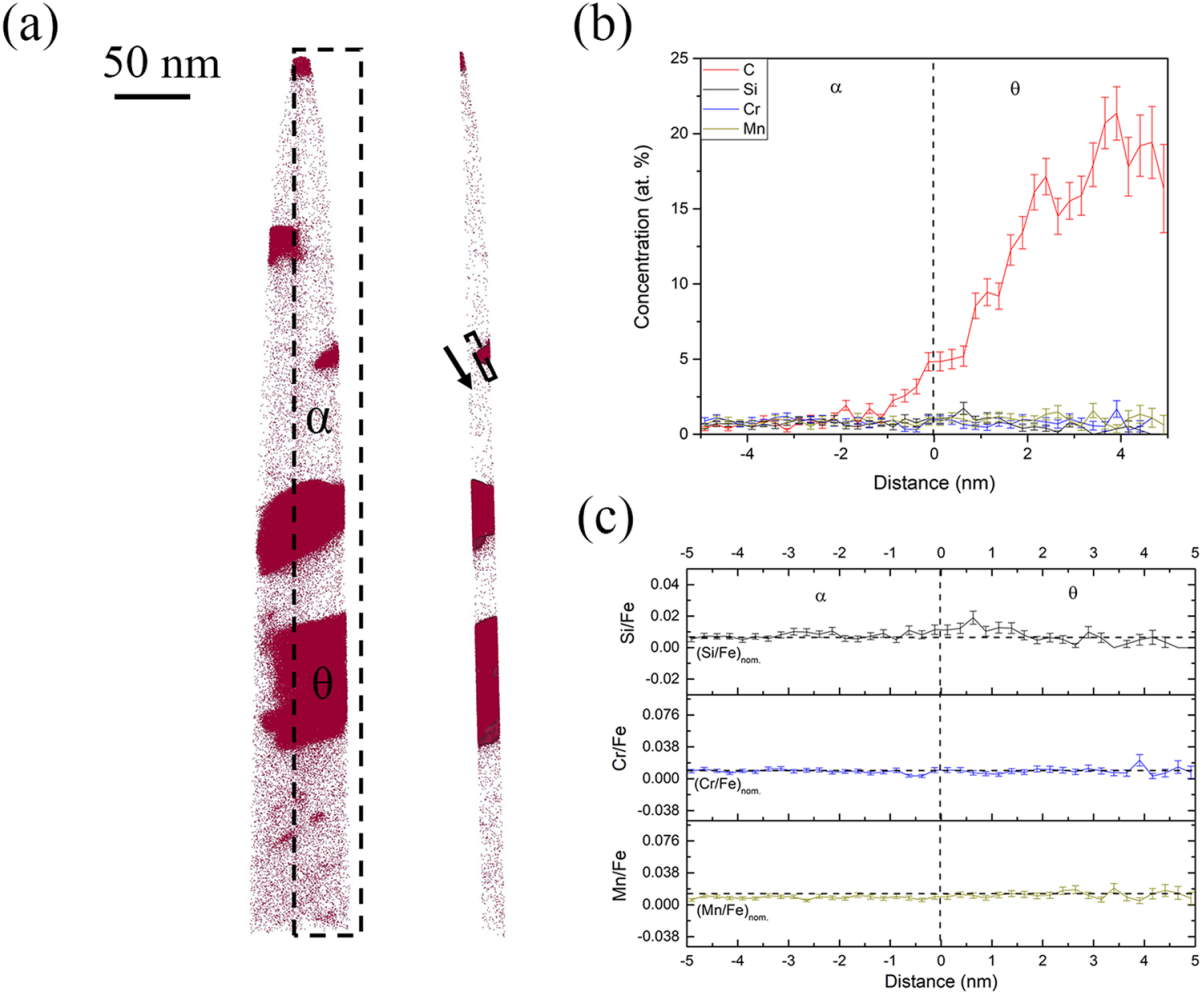
APT reconstructions across in the 5 minute isothermal hold sample showing secondary cementite. (**a**) Atom maps of C within the full needle and a 5 at.% iso-concentration surface from a slice showing the secondary cementite/ferrite interfaces. (**b**) Proximity histogram across the secondary cementite/ferrite interface showing the solute redistribution, (**c**) Ratio of substitutional solute concentration to iron concentration Cr/Fe and Mn/Fe across the secondary cementite/ferrite interface.

**Figure 7:**
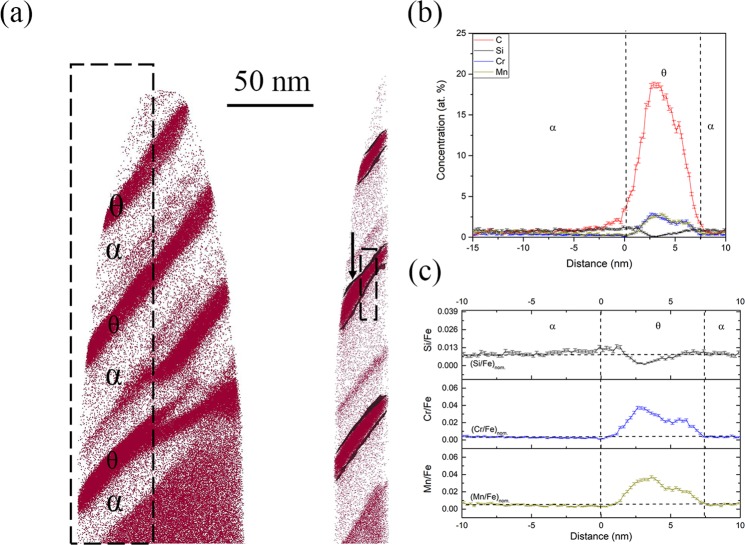
APT reconstructions across of the 10 minute isothermal hold sample showing secondary cementite in ferrite. (**a**) Atom maps of C and 5 at.% iso-concentration surface showing the secondary cementite/ferrite interfaces. (**b**) Proximity histogram across the secondary cementite/ferrite interface showing the solute redistribution, (**c**) Ratio of substitutional solute concentration to iron concentration Cr/Fe and Mn/Fe across the secondary cementite/ferrite interface.

To characterize the solute redistribution during the secondary carbide growth, the 5 minutes, and 10 minutes isothermal hold samples were characterized for carbides within inverse bainitic ferrite. Figures 6 and 7 display the APT results for the 5 minutes and 10 minutes isothermal hold samples respectively. Figure 6a shows the carbon atom map and the 5 at.% iso-concentration surface showing the different secondary carbide/inverse bainitic ferrite interface. Representative proximity histogram across one of the secondary carbide/ferrite interface is shown in Fig. 6b. It can be seen that carbon redistributes between the inverse bainitic ferrite and secondary carbide. The carbon concentration in the secondary carbide is around 20 at.%, indicating that the carbide is cementite. Carbon concentration is lower in inverse bainitic ferrite (approx. 0.227 at.%). No redistribution of Cr, Mn, or Si is observed. The ratio of substitutional solutes (Cr, Mn, and Si) to iron atoms is shown in Fig. 6c. Figure 6c confirms that the concentration of substitutional solutes is preserved across the interface, and no long range diffusion of substitutional solutes take place across the transformation interface. Figure 7a shows the carbon atom map and the 5 at.% iso-concentration surface showing the secondary cementite/inverse bainitic ferrite interfaces when the isothermal holding time is increased to 10 minutes. Figure 7b shows the representative proximity histogram across one of the secondary cementite/inverse bainitic ferrite interface. From Fig. 7b, it can be seen that carbon redistributes across the secondary cementite/inverse bainitic ferrite interface. The carbon concentration in the carbide is around 20 at.% indicating that the carbide is cementite. The carbon concentration is ferrite is low (0.12 at.%) and close to the para-equilibrium carbon concentration in ferrite. It can also be seen that there is Cr/Mn enrichment and Si depletion at both of the cementite/inverse bainitic ferrite interfaces. The concentration of Cr, Mn, and Si appears to have reached the nominal concentration away from the cementite/inverse bainitic ferrite interface. In order to verify that equilibrium is established with respect to the substitutional solutes away from the cementite/ferrite interfaces, the ratio of substitutional solutes to iron were calculated and plotted in Fig. 7c. Figure 7c confirms that the concentration of substitutional solutes reaches the nominal value away from both the cementite/ferrite interfaces and no long range diffusion of substitutional solutes take place.

## Discussion

### Si concentration spike at the interface and growth kinetics during inverse bainitic transformation

It can be seen from Figs 2 and 3 that Si concentration spike is observed at the cementite midrib/martensite interface and the cementite midrib/ferrite interface. The amount of Si in cementite in Fig. 2 is almost 0 at.% but the amount of Si in cementite in Fig. 3 is 0.11 at.%. Because the transformation time is increased for the sample shown in from Fig. 2 compared to that shown in Fig. 3, one would expect the Si concentration in cementite to be the sample value of 0 at.% as the cementite midrib grows. It can also be seen that in Fig. 4, a Si concentration spike at the ferrite/martensite interface is observed, but no Si spike is observed at the ferrite/austenite interface in Fig. 5. Because the transformation time is higher for the sample in Fig. 5 compared to that in Fig. 4, one would expect the concentration of Si at the interface to have increased with the growth of ferrite, but no Si partitioning is observed in Fig. 5. This behavior of Si at the transformation interface will be discussed in the following section.

#### Cementite midrib growth kinetics

It can be seen from Fig. 1a,b, that the inverse bainitic cementite midrib nucleates along the prior austenite grain boundary, marked by the red arrow. Thus the analyzed interface in Fig. 2 is a prior austenite grain boundary/cementite midrib interface, whereas in Fig. 3 is the cementite/ferrite interface and cementite/prior austenite (martensite) interfaces are present. Considering the heat treatment used in the current study, additional point defects may be introduced at the prior austenite grain boundaries during the quenching following the austenitization^25^. These point defect can act as solute sinks, thereby forming solute-vacancy complexes. This phenomenon was reported as thermally induced non-equilibrium segregation. Since the atomic radius of silicon is 3 times smaller than Cr, Mn, and Fe, Si has a much higher and a greater tendency to segregate at the prior austenite grain boundary defects. Apart from the mobility of silicon being higher, Si also has higher thermodynamic potential to segregate along the prior austenite grain boundaries at 500 as shown in ref.^25^. Thus, it can be hypothesized that Si concentration spike in Fig. 2 is a consequence of the prior austenite grain boundary segregation rather than solute redistribution during the growth of cementite midrib. This hypothesis is further supported by the B and Cu atom maps in Figs 2 and 3, where B and Cu segregation (B, and Cu are reported as prior austenite grain boundary segregation elements during thermally induced non-equilibrium segregation^26^) is also observed at the prior austenite prior austenite grain boundary (marked by red arrow in Fig. 2). Faulkner^27^ and Wu^28^ have reported that when an elemental spike due to the thermally induced non-equilibrium segregation occurs, a solute depletion trough is observed ahead of the prior austenite grain boundary. Thus, the cementite midrib forms from austenite with a lower Si concentration ahead of the prior austenite grain boundary and not the equilibrium Si concentration of 0.5 at.%. From the above mentioned segregation behavior of Si to the prior austenite prior austenite grain boundaries, the “true” growth kinetics of cementite can only be characterized by analyzing the cementite midrib/ferrite interface in Fig. 3. In Fig. 3, no partitioning of Cr or Mn is observed across the cementite/ferrite interface. A Si concentration spike is observed at the cementite/ferrite interface, and the concentration of Si in cementite is much higher than the equilibrium solubility of Si in cementite. Since an elemental spike usually indicates that negligible partitioning local equilibrium is reached at the interface, and the concentration of Si in cementite being much higher than the partitioning local equilibrium (PLE) prediction (0 at.%) it can be suggested that the cementite midrib growth follows NPLE type of growth kinetics and during the nucleation stage, the cementite midrib might have formed in a true PE type nucleation.

#### Inverse bainitic ferrite growth kinetics

It can be seen that in Fig. 4, the analyzed interface is ferrite/martensite interface, whereas in Fig. 5, the analyzed interface is ferrite/prior austenite interface. It can be seen from the 5 at.% iso-concentration surface in Fig. 4 that close to the ferrite/martensite interface, a carbide particle is observed. Thus, the analyzed proximity histogram has more than one interface. Miller^29^ has reported that when the proximity histogram has more than one interface, an average concentration will be obtained. Thus, it can be hypothesized that the Si spike in Fig. 4, and the high carbon concentration of martensite (7 at.%) closer to the interface is due to the cementite particle in martensite closer to the inverse bainitic ferrite/martensite interface. This hypothesis is further supported by the Si atom map in Fig. 4, where no enrichment of Si at the inverse bainitic ferrite/martensite interface is observed. Thus, it can be suggested that the inverse bainitic ferrite follows a true PE type growth kinetics at the initial stage of the transformation. When the transformation time is increased, Cr/Mn concentration spike is observed (Fig. 5). It should be noted that the concentration of Cr spike is approximately 4.5 at.% for both Cr/Mn which agrees with the concentration described by NPLE thermodynamics (4.36 at.% for Cr and 5.11 at.% Mn), therefore it can be concluded that the kinetics of growth changes from PE to NPLE.

#### Inverse bainitic secondary cementite growth kinetics

Solute redistribution during the growth of secondary cementite are shown in Figs 6 and 7. From Fig. 6, it can be seen that no substitutional solute partitioning is observed across the secondary cementite/ ferrite interface, and the ratio of substitutional solutes is preserved across the interface. Carbon partitioning is observed across the cementite/ferrite interface. Since no substitutional solute partitioning is observed, and the ratio of substitutional solutes is preserved across the interface, it can be concluded that the growth of secondary cementite follows PE type kinetics when the transformation time is shorter. When the transformation time is increased, Cr, Mn, and Si concentration spike is observed at the cementite/ferrite interfaces in Fig. 7. The concentration of the Cr/Mn spike is much lesser than the PLE concentration (6.5 at.% for Cr and 5.5 at.% for Mn). Also, the ratio of substitutional solutes is preserved across the cementite/ferrite interfaces. Therefore, it can be concluded that the secondary cementite growth kinetics transitions from PE to NPLE when the transformation time is increased because the concentration spike occurs at the cementite/ferrite interfaces, while the ratio of substitutional solutes to iron is preserved across the interface.

### Transition in kinetics from PE to NPLE

**Figure 8:**
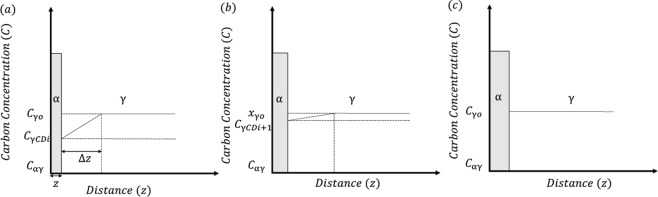
Schematic of the carbon concentration evolution at the ferrite/austenite interface during the growth of inverse bainitic ferrite. (**a**) Ferrite nucleation occurs from carbon depleted austenite, (**b**) As ferrite grows, carbon concentration of austenite increases at the interface, (**c**) Growth instant where the carbon concentration of austenite closer to the ferrite/austenite interface reaches the nominal carbon concentration.

In order to understand the reason for the change in growth kinetics from PE to NPLE, the ferrite/martensite (parent austenite) interface velocity was compared with the critical interface velocity required for the transition in growth kinetics from PE to NPLE. Since the inverse bainitic ferrite growth occurs in a pure PE mode when the transformation time is shorter (only carbon redistribution across the ferrite/austenite interface), the evolution of carbon concentration across the ferrite/austenite interface can be schematically represented as shown Fig. 8. From Fig. 8a, it can be seen that the inverse bainitic ferrite nucleates from carbon depleted austenite (*C*_*γCD*_). The carbon depletion in austenite is caused by the nucleation of cementite, which depletes the carbon concentration of austenite in the vicinity of the cementite/austenite interface. Once the inverse bainitic ferrite nucleates and grows, carbon is rejected into the austenite at the austenite/inverse bainitic ferrite interface. The growth of ferrite increases the carbon concentration in austenite at the austenite/ferrite interface (Fig. 8b), and eventually the carbon concentration of austenite at the austenite/ferrite interface reaches the nominal carbon concentration (Fig. 8c).

Using the principles of diffusion, Zener’s linear approximation of the interface composition profile^30^, the velocity of the inverse bainitic ferrite/austenite interface (*v*_*i*_) at time (*t*_*i*_) can be estimated as

1 $${v}_{i}=\frac{1}{2}\frac{({C}_{\gamma o}-{C}_{\gamma CDi})}{({C}_{\gamma CDi}-{C}_{\alpha \gamma })}\sqrt{\frac{D}{{t}_{i}}}$$

where ${C}_{\gamma o}$ is the bulk/nominal carbon concentration of austenite, ${C}_{\gamma CDi}$ is the initial carbon concentration of the carbon depleted austenite at the ferrite/austenite interface which increments with every time step *t*_*i*_, and ${C}_{\alpha \gamma }$is the carbon concentration of ferrite in equilibrium with austenite. The value of ${C}_{\gamma CDi}$ at time instant *t*_*i*_ can be estimated using the isothermal dilation curves using the expressions

2 $$\begin{array}{rcl}{C}_{(i+1)} & = & {C}_{i}-\frac{5.83}{{(1-{f}_{i})}^{2}}({f}_{(i+1)}-{f}_{i})\,{\rm{and}}\\ {C}_{(i+1)} & = & \frac{[1-[\frac{1}{\frac{({L}_{(i+1)}-{L}_{i})-B({f}_{\gamma ((i+1)}-{f}_{\gamma i})}{A({f}_{\gamma (i+1)}-{f}_{\gamma i})}}+\frac{1}{1-0.0146{C}_{i}}]]}{0.0146}\end{array}$$

Detailed derivation of the above equations are given in Appendix. Once the velocity of the inverse bainitic ferrite/austenite interface is calculated using Equation 1, the velocity is compared with the critical velocity required for conversion to NPLE as reported by Inden in ref.^31^ as:

3 $${v}_{crit}=\frac{\bar{x}}{{t}_{crit}}$$

where $\bar{x}$ is taken as inter-atomic spacing corresponding to four BCC unit cells^32^, and ${t}_{crit}$ can be calculated as the time for diffusion of Mn (substitutional solute with slowest diffusivity) over $\bar{x}$ as

4 $${t}_{crit}=\frac{\overline{{x}^{2}}}{2{D}_{\alpha }^{Mn}}$$

**Figure 9:**
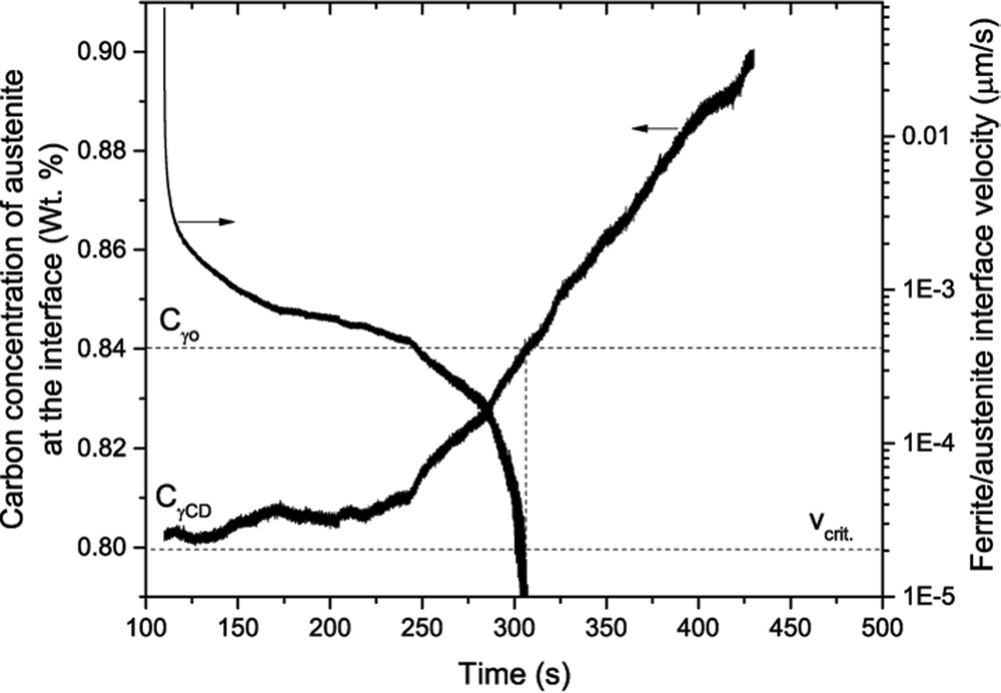
Change in carbon concentration and ferrite/austenite interface velocity with the transformation time. Carbon concentration was calculated from the dilatometry data using Equation 2, interface velocity was calculated using 1, and the critical velocity for PE to NPLE transition was calculated using 3.

The results of the experimental interface velocity calculated using Equations 1 and 2, compared with the critical velocity for PE to NPLE transition is shown in Fig. 9. It can be seen that the carbon concentration of the austenite at the ferrite/austenite interface increases from an initial carbon depletion (${C}_{\gamma CDi}$) of approx. 0.8 wt.% to around 0.9 wt.% towards the end of the transformation. The initial reduction in the carbon concentration at the ferrite/austenite interface is on account of the inverse bainitic cementite midrib formation. It can also be seen that the velocity of the the ferrite/austenite interface decreases with the increase in the transformation time. The reduction in the interface velocity is on account of the buildup of carbon at the ferrite/austenite transformation front. The experimental interface velocity calculated using Equation 1 drops below the theoretically calculated velocity required for Mn diffusion across the ferrite/austenite interface when the transformation time is greater than 310 s. Thus, when the transformation time is increased, the reduction in the interface velocity allows for redistribution of the substitutional solutes with local equilibrium being maintained at the ferrite/austenite interface through the development of a substitutional solute concentration spike at the interface. This explain why the 5 minutes isothermal hold sample shows a pure PE growth kinetics at the ferrite/martensite interface and the kinetics changes to NPLE when the transformation time is increased.

Mesquita^33^ have studied cementite precipitation in low silicon steels and have found that if the early cementite precipitation is not inhibited by Si, the alloying elements do not have carbon to form alloy carbides, but the diffusion distance of alloying elements increases when the transformation time is increased. Therefore the alloying elements like Cr/Mn diffuse into cementite and stabilize it thereby retarding its growth, rather than the dissolution of cementite to form alloy carbides. This reported behaviour in low Si steels is consistent with our observations, where the early secondary cementite precipitation in ferrite is not inhibited by Si (Fig. 6) and Cr and Mn partitioning into cementite is observed at longer transformation times (Fig. 7). Similar transition in kinetics of the cementite growth in ferrite was reported by Caballero^23,24^ during tempering of nanocrytalline bainitic steel. Thus it can be concluded that the non-availability of carbon atoms to form alloy carbides and the increased diffusion of alloy carbides as the transformation time is increased explains why the secondary cementite growth kinetics change from pure PE to NPLE when the transformation time is increased.

**Figure 10:**
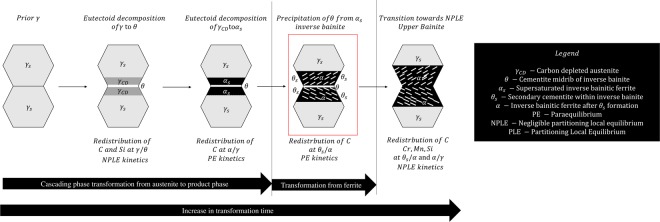
Summary of cascading reactions from supersaturated parent austenite leading to the formation of inverse bainite microstructure. The red box represents the microstructure of inverse bainite formed as a result of individual cascading reactions from parent austenite and supersaturated ferrite.

The APT/SEM results presented in the study provide a strong indication that the inverse bainitic transformation occurs as a consequence of individual cascading phase transformations starting from parent austenite. The cascading reaction theory is assumed throughout the discussion on how diffusion controls the reactions by assessing kinetic compatibility. When parent austenite is quenched to the isothermal hold temperature, metastable cementite is nucleated as a consequence of higher thermodynamic driving force. This results in the redistribution of carbon across the parent austenite cementite interface. When the transformation time is increased, Si redistributes across the parent austenite cementite interface along with carbon resulting in a NPLE type of growth kinetics. The partitioning of carbon from parent austenite into cementite locally reduces the carbon concentration of austenite, thereby increasing the driving force for ferrite formation, resulting in the nucleation of ferrite at the cementite/carbon depleted austenite interface. During the ferrite nucleation and growth, there is redistribution of carbon without any redistribution of substitutional solutes at the ferrite/austenite interface. Apart from the above mentioned cascading reactions from parent austenite, secondary cementite particles nucleate within ferrite by redistribution of carbon at the ferrite/cementite interface. Thus, the combination of cascading phase transformations from parent austenite and the nucleation of cementite from ferrite results in the formation of inverse bainitic microstructure. When the transformation time is increased further, the para-equilibrium microstructure of inverse bainite transitions towards an negligible partitioning local equilibrium structure by partitioning of substitutional solutes at the interfaces, resulting in the formation of the degenerated microstructure of inverse bainite (upper bainite). It is likely that when the transformation time is increased beyond the formation of degenerated microstructure, auto-tempering of bainite will occur resulting in gradual transition of NPLE - PLE as reported by Caballero^23^. The different cascading phase transformations and the corresponding elemental partitioning behaviour are schematically shown in Fig. 10. Results provide an indication that inverse bainitic transformation proceeds in a similar manner to Widmanstätten ferrite/bainitic ferrite with carbon diffusion controlled growth, and without any reconstructive or long range diffusion of substitutional solutes.

## Conclusion

Solute redistribution during isothermal transformation of austenite to inverse bainite have been characterized at atomic scale for the first time. The APT results presented in the present study on the growth kinetics of inverse bainite provide a strong indication that the inverse bainitic transformation occurs as a consequence of individual cascading phase transformations starting from parent austenite. Solute concentration profiles at different transformation interfaces indicated growth kinetics, and the following conclusions were made:

1. Cementite midrib of inverse bainite follows NPLE type growth kinetics. Though exact nucleation stage of the cementite midrib could not be characterized, it is likely that the cementite midrib nucleation occurs in a PE mode.
2. Inverse bainitic ferrite nucleation occurs in a PE mode, and when the transformation time is increased, there is a transition in kinetics from PE to NPLE. Interface velocity calculations showed that a deviation from PE occurs when the carbon concentration of the austenite at the interface reaches the bulk carbon concentration, by when the velocity of the interface drops below the critical velocity required for substitutional solute diffusion. The reduction in the interface velocity due to the carbon enrichment at the interface is the likely reason for the transition in kinetics from PE to NPLE.
3. Secondary cementite within inverse bainitic ferrite nucleate in a PE mode and when the transformation time is increased, there is a transition in kinetics from PE to NPLE.

The results provide an indication that inverse bainitic transformation occurs in a similar manner to Widmanstätten ferrite/bainitic ferrite with carbon diffusion controlled growth, and without any reconstructive diffusion.

## Experimental Methods

### Isothermal heat treatments

Isothermal bainite transformation were conducted on a hypereutectoid (roughly 4 at.% carbon) steel alloyed with 1 at.% each of Cr and Mn as the major alloying additions and trace of silicon (approx. 0.4 at.%). Considering the narrow temperature range for inverse bainitic transformation in the steel used in the present study^34^, a temperature of 500 °C was used to carry out isothermal bainite transformation. A RITA L78 high speed quench dilatometer was used to heat the samples to an austenitization temperature of 1050 °C in order to render a homogeneous carbon/alloying element distribution in austenite followed by cooling the sample to an isothermal holding temperature of 500 °C. Samples were held at 500 for 1 minute, 1.5 minutes, 3 minutes, 5 minutes, 7 minutes, and 10 minutes followed by quenching to room temperature. Detailed microstructural analysis and dilation curve analysis for each of the heat treatment were conducted and can be found in refs^20,35^.

### Atom Probe Tomographt (APT)

Site-specific needles for APT analysis were extracted from the 1 minute (cementite midrib formation), 3 minutes (inverse bainitic ferrite formation), 5 minutes (inverse bainitic ferrite and secondary carbide formation), and 10 minutes (degeneration of inverse bainite) isothermal hold samples using a FEI Nova 200 dual beam FIB/SEM^36^. Regions of interest were annularly milled and cleaned at low voltage (2 kV) with a Ga ion beam to make the needle shaped specimens suitable for field evaporation. Atom probe analyses were performed using a CAMECA Instruments LEAP 4000X HR local electrode atom probe (LEAP). The atom probe needles were field evaporated in laser mode with a 200 kHz pulse repetition rate, 35 K specimen temperature, 50–70 pJ laser power, and a detection rate of 0.5%. The resulting data was reconstructed and analyzed using CAMECA’s IVAS software.

## Data Availability

The authors declare that the data supporting the findings of this study are available within the paper.

## Appendix

**Estimation of local carbon concentration in austenite using dilation curves**

The meaning of main symbols along with their units used in the equations below are shown in Table 1.

**Table 1:** Nomenclature of symbols used.

Symbol	Name	Units
*V*	Total volume of the sample	*µm* ^3^
*m*_*T*_	Total mass of the sample	*gm*
*V*_*o*_	Initial volume of the sample	*µm* ^3^
*ρ*_*o*_	Initial density of the sample	*gmµm* ^*−*3^
*ρ*_*i*_	Density of constituent *i*	*gmµm* ^*−*3^
*V*_*i*_	Volume of constituent *i*	*µm* ^3^
*λ*_*i*_	Ratio of density of sample to density of the phase $(\frac{{\rho }_{o}}{{\rho }_{i}})$	
*ρ*_*γ*( *T*, *C*)_	Density of austenite at temperature *T* and carbon content *C*	*gmµm* ^*−*3^
*ρ*_*γ*( *T*, *C*′)_	Density of austenite at Temperature *T* and carbon content after primary cementite transformation	*gmµm* ^*−*3^
*C*_*γ*_	Carbon content of austenite	*Wt*.%
*C*_*θ*_	Equilibrium carbon content of cementite	*Wt*.%
*f*_*γ*_	Fraction of austenite	
*f*_*θ*_	Fraction of cementite	
*f*_*α+θ*_	Fraction of ferrite + cementite	

**Carbon content estimation during cementite formation from parent austenite**

The volume of specimen during the transformation is given by

$$\gamma \to {\gamma }_{depleted}+{\theta }_{primary}$$

$$V=\sum {V}_{i}$$

$$V=\sum \frac{{m}_{T{f}_{i}}}{{\rho }_{i}}$$

$$V={V}_{o}\sum \frac{{\rho }_{o{f}_{i}}}{{\rho }_{i}}$$

$$\frac{V}{{V}_{o}}=\sum {\lambda }_{i}\,{f}_{i}$$

For the decrease portion of dilatation, i.e. cementite formation from austenite, there is carbon redistribution. The carbon content of the parent austenite decreases due to the formation of austenite, because of which there is a decreased dilatation during the initial formation of cementite. Writing the above Equation for the formation of cementite from austenite,

$$\frac{V}{{V}_{o}}={\lambda }_{\gamma }\,{f}_{\gamma }+\,{\lambda }_{\theta }\,{f}_{\theta }$$

$$\frac{V}{{V}_{o}}={\lambda }_{\gamma }-\,{\lambda }_{\gamma }\,{f}_{\theta }+{\lambda }_{\theta }\,{f}_{\theta }={\lambda }_{\gamma }+{f}_{\theta }({\lambda }_{\theta }\,-\,{\lambda }_{\gamma })$$

Since the experiments are performed in isothermal holding condition, differentiating with respect to time gives,

5 $$\frac{1}{{V}_{o}}\frac{dV}{dt}=\frac{\partial {\lambda }_{\gamma }}{\partial t}-{f}_{\theta }\frac{\partial {\lambda }_{\gamma }}{\partial t}+\frac{\partial {f}_{\theta }}{\partial t}({\lambda }_{\theta }\,-\,{\lambda }_{\gamma })$$

The above equation is with the assumption that density of cementite does not vary with carbon concentration. In the above equation ${\lambda }_{\gamma }=\frac{{\rho }_{o}}{{\rho }_{\gamma (T,c)}}$.

6 $$\frac{\partial {\lambda }_{\gamma }}{\partial t}=-\frac{{\rho }_{o}}{{\rho }_{\gamma (T,C)}}\frac{1}{{\rho }_{\gamma (T,C)}}\frac{\partial {\rho }_{\gamma }}{\partial t}=-\frac{{\rho }_{o}}{{\rho }_{\gamma (T,C)}}\frac{1}{{\rho }_{\gamma (T,C)}}\frac{\partial {\rho }_{\gamma }}{\partial {C}_{\gamma }}\frac{\partial {C}_{\gamma }}{\partial t}$$

7 $${\rho }_{\gamma (T,C)}={\rho }_{\gamma (T,C=0)}(1-0.0146 \% C)$$

8 $$\frac{\partial {\rho }_{\gamma (T,C)}}{\partial {C}_{\gamma }}=-\,{\rho }_{\gamma (T,C=0)}0.0146$$

Substituting Equation 7 and Equation 8 in Equation 6,

9 $$\frac{\partial {\lambda }_{\gamma }}{\partial t}=\frac{{\rho }_{o}}{{\rho }_{\gamma (T,C=0)}}\frac{0.0146}{{(1-0.0146 \% C)}^{2}}\frac{\partial {C}_{\gamma }}{\partial t}$$

Substituting Equation 9 in Equation 5,

$$\frac{1}{{V}_{o}}\frac{dV}{dt}=\,\frac{{\rho }_{o}}{{\rho }_{\gamma (T,C=0)}}\frac{0.0146}{{(1-0.0146 \% C)}^{2}}\frac{\partial {C}_{\gamma }}{\partial t}\,(1-{f}_{\theta })+\frac{\partial {f}_{\theta }}{\partial t}{\rho }_{o}[\frac{{\rho }_{\gamma (T,C)}-{\rho }_{\theta }}{{\rho }_{\gamma (T,C)}{\rho }_{\theta }}]$$

**Approximation 1:** The second term in the above equation is negligible and is assumed zero.

10 $$\frac{1}{{V}_{o}}\frac{dV}{dt}=\,\frac{{\rho }_{o}}{{\rho }_{\gamma (T,C=0)}}\frac{0.0146}{{(1-0.0146 \% C)}^{2}}\frac{\partial {C}_{\gamma }}{\partial t}\,(1-{f}_{\theta })$$

Writing mass balance with respect to carbon,

$${C}_{\gamma }{f}_{\gamma }+{C}_{\theta }{f}_{\theta }=0.84$$

$${C}_{\gamma }=\frac{0.84-6.67{f}_{\theta }}{1-{f}_{\theta }}$$

11 $$\frac{\partial {C}_{\gamma }}{\partial t}=-\frac{5.83}{{(1-{f}_{\theta })}^{2}}\frac{\partial {f}_{\theta }}{\partial t}$$

Substituting Equation 11 in Equation 10,

$$\frac{1}{{V}_{o}}\frac{dV}{dt}=\,-\frac{{\rho }_{o}}{{\rho }_{\gamma (T,C=0)}}\frac{0.0146}{{[1-0.0146(\frac{0.84-6.67{f}_{\theta }}{1-{f}_{\theta }})]}^{2}}\frac{5.83}{(1-{f}_{\theta })}\frac{\partial {f}_{\theta }}{\partial t}$$

$$\frac{3}{{L}_{o}}\frac{dL}{dt}=-\frac{{\rho }_{o}}{{\rho }_{\gamma (T,C=0)}}\,0.0146\,\frac{5.83}{(1-{f}_{\theta })}\frac{\partial {f}_{\theta }}{\partial t}$$

$$\frac{dL}{dt}=\frac{{\rho }_{o}{L}_{o}0.08511}{3{\rho }_{\gamma (T,C=0)}}\frac{\partial (\mathrm{ln}(1-{f}_{\theta }))}{\partial t}$$

Which leaves an Euler type integration scheme as below,

$$\frac{{L}_{2}-{L}_{1}}{K}=ln[\frac{1-{f}_{2}}{1-{f}_{1}}]$$

Which on further simplification leaves,

12 $${f}_{i+1}=1-[(1-{f}_{i})\exp (\frac{{L}_{i+1}-{L}_{i}}{K})]$$

Where

$$K=\frac{{\rho }_{o}{L}_{o}0.08511}{3{\rho }_{\gamma (T,C=0)}}$$

Thus, once the phase fraction of initial cementite nuclei is obtained from raw dilatometry data, the fraction can be substituted in Equation 10 to leave an Euler integration scheme for carbon content in austenite as below:

13 $${C}_{i+1}=\,{C}_{i}-\frac{5.83}{{(1-{f}_{i})}^{2}}({f}_{i+1}-{f}_{i})$$

**Carbon content estimation during inverse bainitic ferrite transformation**

For the formation of inverse bainitic ferrite, we can write equation similar to the formation of cementite discussed in the previous section

$$\frac{V}{{V}_{o}}=k{\lambda }_{\gamma }+{f}_{\alpha +\theta }({\lambda }_{\alpha +\theta }-{\lambda }_{\gamma })$$

Here *k* is the fraction of austenite left untransformed after stage one of the transformation.

$$\frac{1}{{V}_{o}}\frac{dV}{dt}=k\frac{\partial {\lambda }_{\gamma }}{\partial t}-{f}_{\alpha +\theta }\frac{\partial {\lambda }_{\gamma }}{\partial t}+({\lambda }_{\alpha +\theta }-{\lambda }_{\gamma })\frac{\partial {f}_{\alpha +\theta }}{\partial t}$$

14 $$\frac{1}{{V}_{o}}\frac{dV}{dt}=\frac{\partial {\lambda }_{\gamma }}{\partial t}(k-{f}_{\alpha +\theta })+({\lambda }_{\alpha +\theta }-{\lambda }_{\gamma })\frac{\partial {f}_{\alpha +\theta }}{\partial t}$$

From the previous model in ref.^35^,

15 $$\frac{\partial {\lambda }_{\gamma }}{\partial t}=\frac{{\rho }_{o}}{{\rho }_{\gamma (T,C=0)}}\frac{0.0146}{{(1-0.0146 \% C)}^{2}}\frac{\partial {C}_{\gamma }}{\partial t}$$

Substituting Equation 15 in the Equation 14,

$$\frac{1}{{V}_{o}}\frac{dV}{dt}=\frac{{\rho }_{o}}{{\rho }_{\gamma (T,C=0)}}\frac{0.0146}{{(1-0.0146 \% C)}^{2}}\frac{\partial {C}_{\gamma }}{\partial t}(k-{f}_{\alpha +\theta })+({\lambda }_{\alpha +\theta }-{\lambda }_{\gamma })\frac{\partial {f}_{\alpha +\theta }}{\partial t}$$

$$\frac{3}{{L}_{o}}\frac{dL}{dt}=\frac{{\rho }_{o}}{{\rho }_{\gamma (T,C=0)}}\frac{0.0146}{{(1-0.0146 \% C)}^{2}}\frac{\partial {C}_{\gamma }}{\partial t}(k-{f}_{\alpha +\theta })+({\lambda }_{\alpha +\theta }-{\lambda }_{\gamma })\frac{\partial {f}_{\alpha +\theta }}{\partial t}$$

$$\frac{3}{{L}_{o}}\frac{dL}{dt}=\frac{{\rho }_{o}}{{\rho }_{\gamma (T,C=0)}}(k-{f}_{\alpha +\theta })\frac{\partial [\frac{1}{(1-0.0146 \% {C}_{\gamma })}]}{\partial t}+{\rho }_{o}[\frac{{\rho }_{\gamma }-{\rho }_{\alpha +\theta }}{{\rho }_{\gamma }{\rho }_{\alpha +\theta }}]\frac{\partial {f}_{\alpha +\theta }}{\partial t}$$

$$\frac{dL}{dt}=\frac{{\rho }_{o}{L}_{o}}{3{\rho }_{\gamma (T,C=0)}}(k-{f}_{\alpha +\theta })\frac{\partial [\frac{1}{(1-0.0146 \% {C}_{\gamma })}]}{\partial t}+\frac{{\rho }_{o}{L}_{o}}{3}[\frac{{\rho }_{\gamma }-{\rho }_{\alpha +\theta }}{{\rho }_{\gamma }{\rho }_{\alpha +\theta }}]\frac{\partial {f}_{\alpha +\theta }}{\partial t}$$

$$\frac{dL}{dt}=A(k-{f}_{\alpha +\theta })\frac{\partial [\frac{1}{(1-0.0146 \% {C}_{\gamma })}]}{\partial t}+B\frac{\partial {f}_{\alpha +\theta }}{\partial t}$$

*A* and *B* are material constants given by

$$A=\frac{{\rho }_{o}{L}_{o}}{3{\rho }_{\gamma (T,C=0)}}$$

$$B=\frac{{\rho }_{o}{L}_{o}}{3}[\frac{{\rho }_{\gamma }-{\rho }_{\alpha +\theta }}{{\rho }_{\gamma }{\rho }_{\alpha +\theta }}]$$

Solving for ${C}_{\gamma }$ an Euler type integration scheme which can be used to calculate the carbon content of austenite during the second stage of the transformation, with carbon content of austenite calculated from the first stage as the initial input.

$${C}_{i+1}=\frac{[1-\frac{1}{[\frac{({L}_{i+1}-{L}_{i})-B({f}_{\gamma (i+1)}-{f}_{\gamma i})}{A({f}_{\gamma (i+1)}-{f}_{\gamma i})}+\frac{1}{1-0.0146{C}_{i}}]}]}{0.0146}$$
